# The peristomial plates of ophiuroids (Echinodermata: Ophiuroidea) highlight an incongruence between morphology and proposed phylogenies

**DOI:** 10.1371/journal.pone.0202046

**Published:** 2018-08-09

**Authors:** Iain C. Wilkie, Martín I. Brogger

**Affiliations:** 1 Institute of Biodiversity, Animal Health and Comparative Medicine, University of Glasgow, Glasgow, Scotland, United Kingdom; 2 Laboratorio de Reproducción y Biología Integrativa de Invertebrados Marinos, IBIOMAR-CONICET, Blvd. Almirante Brown, Puerto Madryn, Argentina; Ecole Normale Supérieure de Lyon, FRANCE

## Abstract

The peristomial plates are skeletal components of the interbrachial frame (or mouth frame), which is located below the true mouth of ophiuroids. Whilst the peristomial plates were extensively described and used as diagnostic characters by some early workers, for the past 100 years they have been largely neglected as a taxonomic resource. In this investigation the peristomial plates of 48 species representing 21 families were examined directly, and information on a further 61 species, including representatives of another eight families, was obtained from the published literature. Observations were made with regard to fragmentation state, relative size and orientation of the peristomial plates. Although fragmentation state showed little consistency at any taxonomic level, relative size and orientation segregated a group of families comprising species with relatively small, inclined peristomial plates, viz. Ophiotrichidae, Ophiopholidae, Ophiactidae, Amphiuridae and Ophiocomidae, together with a single hemieuryalid species–*Ophioplocus januarii*. The distribution of peristomial plate traits was strongly correlated with that of several other character states pertaining to the interbrachial frame. This supported the proposition that two major types of interbrachial frame are present in ophiuroids (designated ‘A’ and ‘B’). Current phylogenies inferred from both morphological and molecular data imply that type B is derived and has evolved independently at least twice in the orders Amphilepidida and Ophiacanthida. This represents a remarkable example of evolutionary convergence. An analysis of the distribution of all interbrachial frame character states suggested that within the Amphilepidida paedomorphosis was probably responsible for the complete reversion of the interbrachial frame to the ancestral type A condition in two families (Ophiothamnidae and Amphilepididae) of suborder Gnathophiurina and possibly responsible for varying degrees of trait reversal in the four families of suborder Ophionereidina. Such paedomorphic events may have been associated with a secondary return to the deep-sea from shallow-sea environments.

## Introduction

The Ophiuroidea (brittlestars, snakestars and basketstars) is the largest of the five surviving echinoderm classes in terms of described extant species (>2,000). Ophiuroids are an important and sometimes dominant component of benthic communities from tropical to polar latitudes and from the littoral to the hadal zones [1,2], consequences of which include the abundance of their disarticulated skeletons in most marine sediments and in some micropalaeontological samples [3].

Ophiuroid taxonomy and systematics are currently in a state of flux: the re-evaluation of suprageneric diagnostic characters in use for the past 100 years, the employment in phylogenetic analyses of many new skeletal traits, accumulating evidence for the importance of heterochronic processes, particularly paedomorphosis, and the application of molecular techniques have generated morphological and genomic phylogenies that show impressive congruence and have led to a complete overhaul of ophiuroid classification at the family level and above [2–11]. This revision is likely to continue apace as the datasets of characters and species expand and further molecular analyses are conducted [2,9].

One unsatisfactory aspect of current ophiuroid classificatory schemes and morphology-based phylogenies is that the systematic characters on which they are based are dominated by features of the external skeletal system [3,9]. As observed over 160 years ago by Gaudry [12], the adult ophiuroid endoskeleton comprises *internal* components, i.e. the vertebrae of the arms and the interbrachial frame that links together the vertebral series of all the arms, and *external* components, i.e. the outer plates of the arms, ventral interbrachial regions and central disc, and the appendages attached to these plates, such as arm spines, podial scales and mouth papillae (Gaudry classified the external plates and their appendages as the ‘dermal’ and ‘epidermal’ systems respectively). In a recent phylogenetic analysis, 128 of the 130 morphological characters employed were derived from the endoskeleton: 113 (88%) of these were from the external plates and their appendages and 15 (12%) from the vertebrae and interbrachial frame [6].

**Fig 1:**
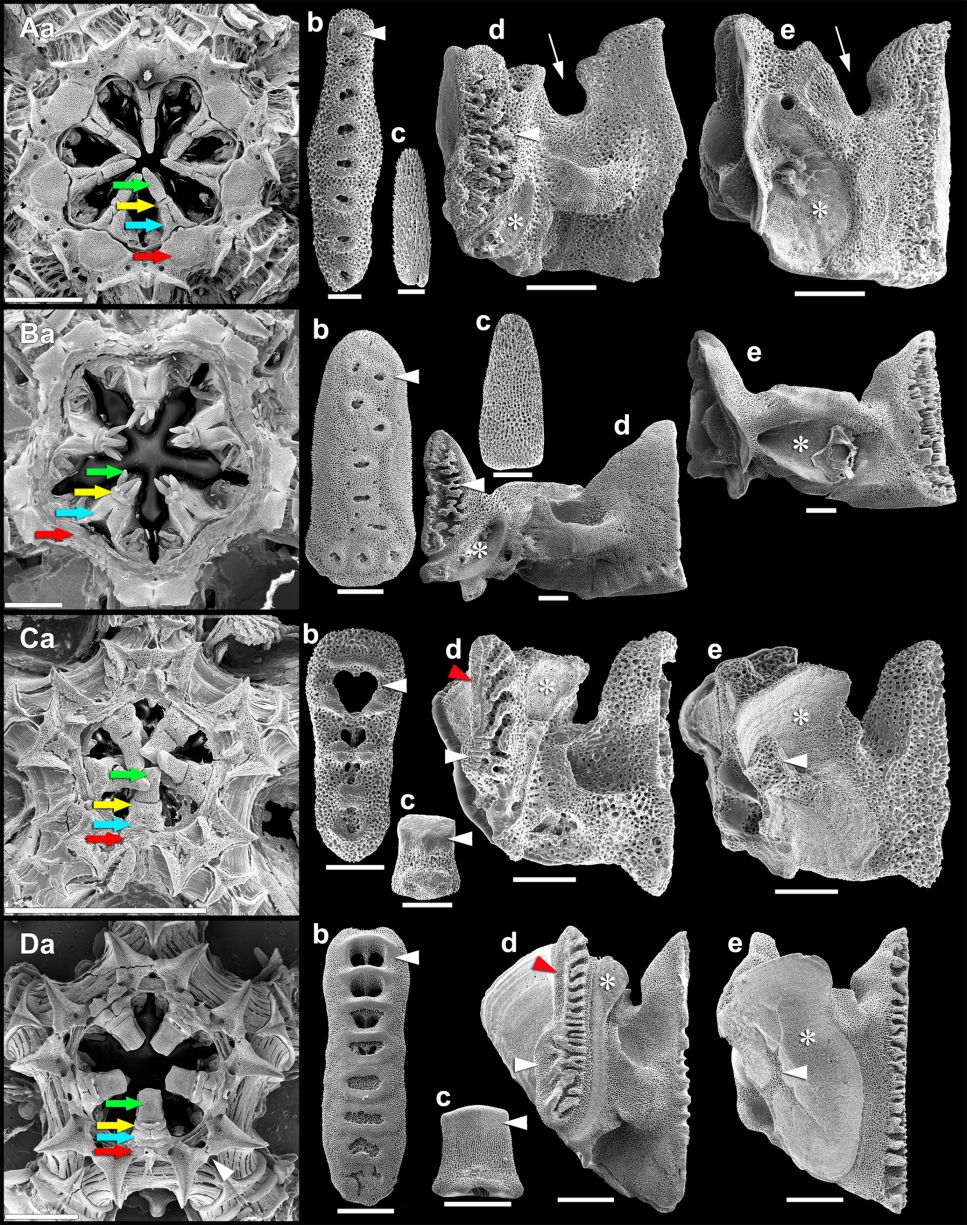
Introduction to the ophiuroid interbrachial frame. Scanning electron micrographs of whole interbrachial frames and their isolated skeletal components of (A) *Ophiacantha vivipara*, (B) *Ophiura lymani*, (C) *Amphiura eugeniae* and (D) *Ophioplocus januarii*. (a) Whole interbrachial frame (aboral side). Arrows: green, tooth; yellow, dental plate; blue, oral plate; red, peristomial plate. White arrowhead (in Da), advertebral groove. (b) Dental plate (proximal side, aboral end at top). Arrowhead, tooth socket. (c) Tooth. Arrowhead, cap of imperforate (*A*. *eugeniae*) or more densely fenestrated (*O*. *januarii*) stereom. (d) Oral plate (adradial side, aboral end at top). Arrow (in Ad), neural groove; white arrowhead, adradial articular area; red arrowhead, advertebral groove (showing attachment area of distal radial ligament); asterisk, adradial muscle attachment area. (e) Oral plate (abradial side, aboral end at top). Arrow (in Ae), neural groove; arrowhead, interruption of differentiated stereom associated with bursal diverticulum; asterisk, abradial muscle attachment area. Scalebars: 2 mm (Aa, Ba, Ca, Da); 0.5 mm (Ad,e, Bb-e, Db-e); 0.2 mm (Ab,c, Bc, Cb-e).

**Fig 2:**
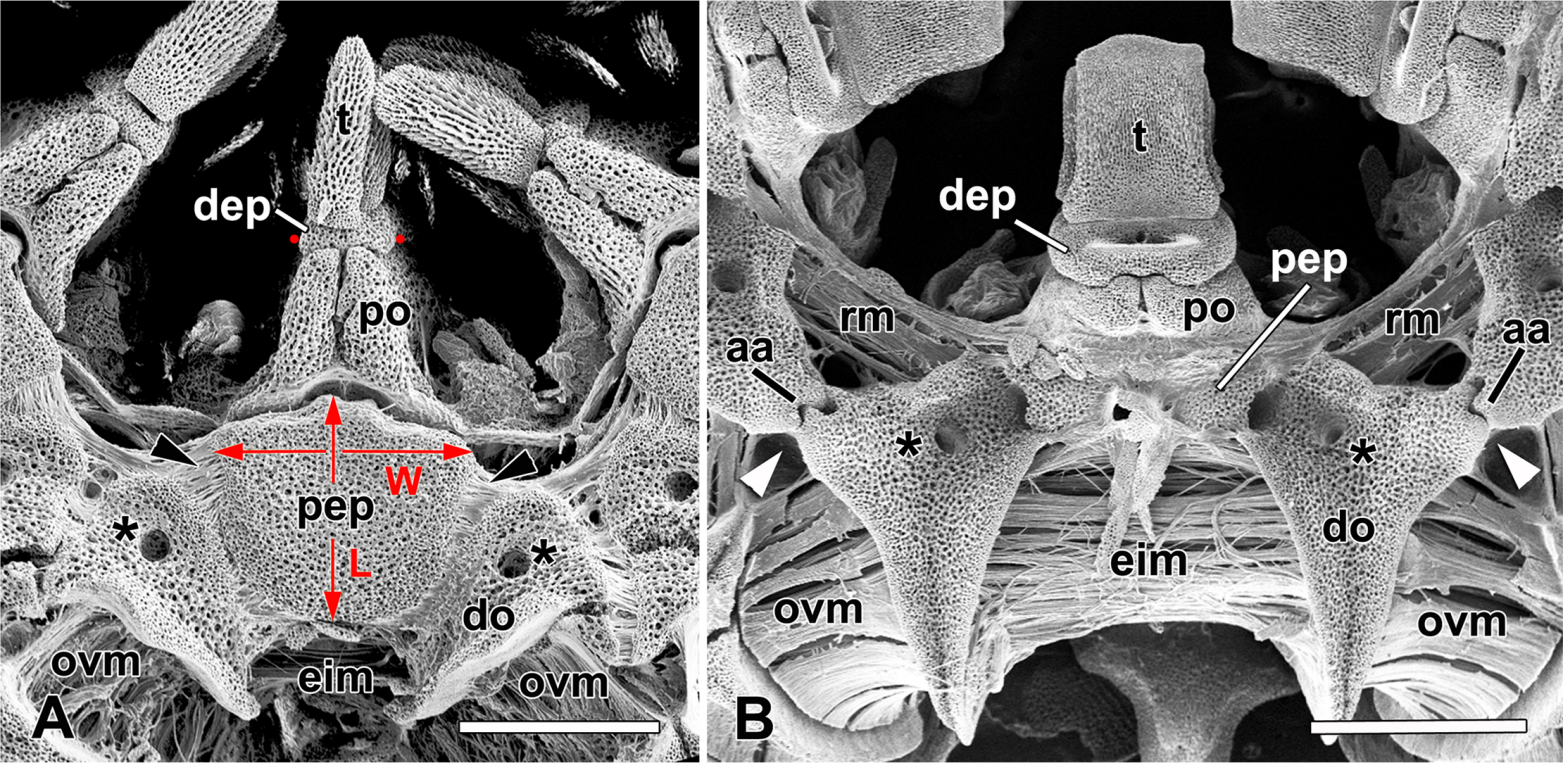
**Scanning electron micrographs showing aboral side of a single interbrachial frame unit (“jaw”) of (A) *Ophiacantha vivipara* (see Fig 1Aa) and (B) *Ophioplocus januarii* (see Fig 1Da).** aa, adradial articulation; dep, dental plate; do, distal part of oral plate; eim, external interradial muscle (between abradial sides of adjacent oral plates); L, maximum length of peristomial plate; po, proximal part of oral plate; ovm, orovertebral muscle (between oral plate and first vertebral ossicle); pep, peristomial plate; rm, radial muscle (between adradial sides of adjacent oral plates); t, tooth; arrowhead, ligament connecting peristomial plate to oral plate; W, maximum width of peristomial plate; asterisk, pore for water vascular canal that connects circumoral water vascular ring to buccal podia; red dots, lateral edges of aboral region of dental plate, the distance between which is the maximum transverse width, as defined herein. Scalebars: 1 mm.

**Fig 3:**
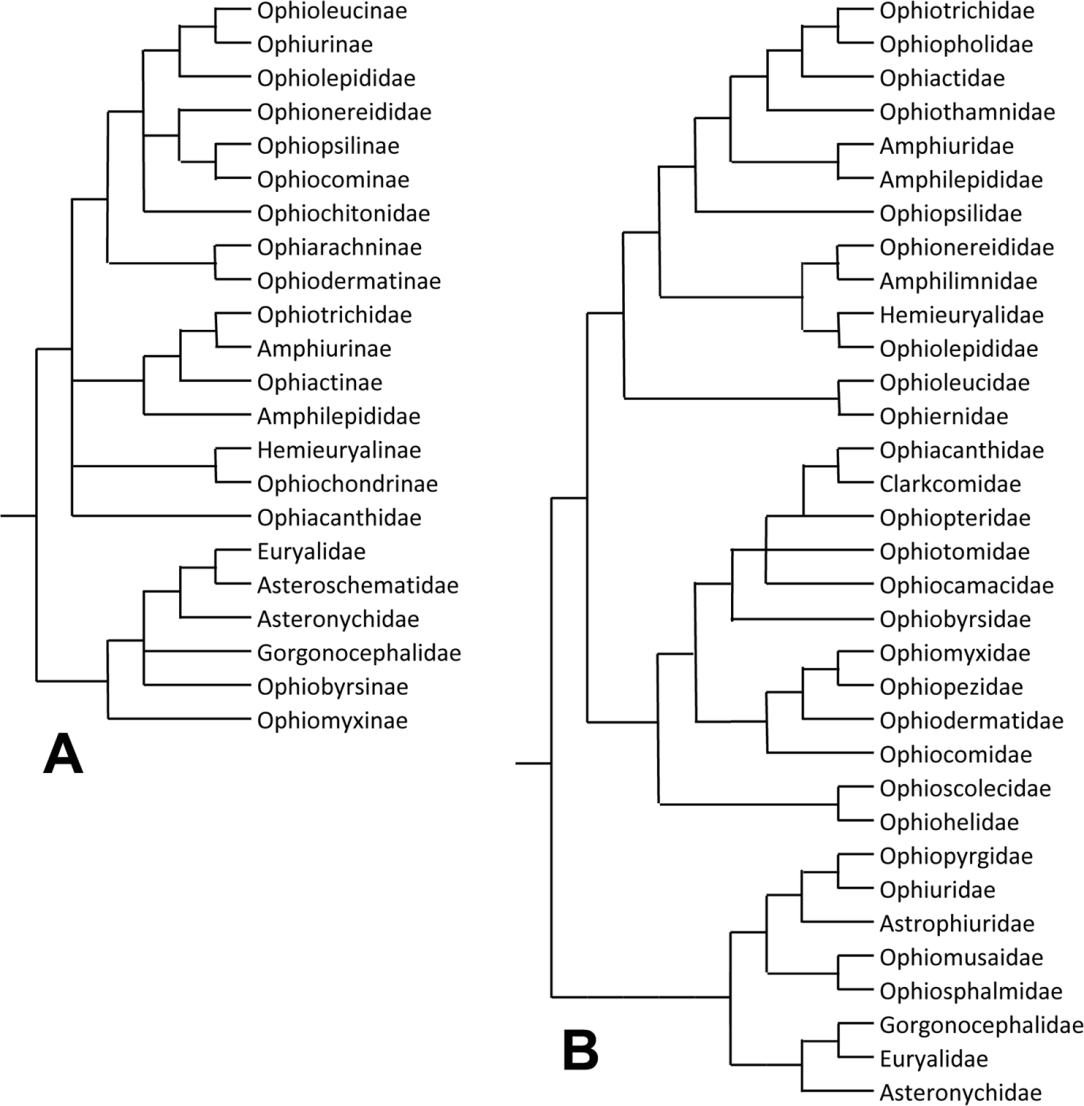
Old and new phylogenetic trees of class Ophiuroidea. (A) Cladogram derived by Smith et al. [23] from the phylogenetic diagrams of Matsumoto [21] (slightly modified). (B) Cladogram derived from the phylogenetic tree of O’Hara et al. [9] (slightly modified).

The skeletal components of the interbrachial frame (IF) are the teeth, dental plates, oral plates and peristomial plates (Figs 1 and 2). Although the IF has previously been referred to in different languages as, *inter alia*, the “mouth-skeleton”, “masticatory apparatus”, “jaw apparatus”, “mouth-frame” and “oral frame”, the less functionally presumptive “interbrachial frame” is introduced herein because there is no empirical evidence that either (1) the main functions of the IF or (2) the main evolutionary drivers influencing its morphology are related to mastication or any other aspect of alimentation (see ‘Discussion‘). IF components, particularly the dental and oral plates and their soft tissue correlates, were accurately described and their mechanical functioning speculated upon by several 19th century biologists [13–19]. However, Matsumoto [20,21] was the first to exploit their taxonomic potential by including features of the oral plates and peristomial plates in his diagnoses of extant families and orders. He also identified an incongruence between IF morphology and his proposed phylogeny of the Ophiuroidea. In several places in his text [21] he drew attention to IF character states that were peculiar to the families Amphiuridae (*sensu* Matsumoto, i.e. Amphiurinae + Ophiactinae), Ophiotrichidae and Ophiocomidae (*sensu* Matsumoto, i.e. Ophiocominae + Ophiopsilinae), the subfamily Ophionereidinae (currently subsumed within the Ophionereididae together with the former subfamily Ophiochitoninae) and the single species *Ophioceramis januarii* (Lütken, 1856) (which was then in the family Ophiolepididae, subfamily Ophiolepidinae and which was subsequently renamed *Ophioplocus januarii* and assigned to the Hemieuryalidae *sensu* O’Hara et al. [2,9,22]). Matsumoto observed that these taxa all have robust, square- or blunt-ended teeth, oral plates with very large attachment areas (which he called ‘wings’) for the external interradial muscles, and small peristomial plates. However, he acknowledged that, according to his proposed phylogeny, this combination of traits, which he assumed were derived, had “developed more or less independently in three lines” ([21], p. 382), these being (1) Amphiuridae + Ophiotrichidae, (2) Ophiocomidae + Ophionereidinae and (3) *Ophioceramis januarii* (Fig 3A).

Murakami [24] provided detailed information on the morphology of the dental and oral plates of 120 species, almost all of which were illustrated in line drawings. Whereas Matsumoto [21] had commented on, and illustrated, only the dorsal side of the oral plates, Murakami focused on their lateral (abradial and adradial) sides, thus making available a wider set of features with potential systematic value. Although Murakami’s data revealed more IF character states found only in Matsumoto’s lines 1 and 2 (*Ophioceramis/Ophioplocus januarii* was not included in his investigation), his phylogenetic tree, which was based on Matsumoto’s, still implied homoplasy rather than homology. On the basis of a short review of the information then available on all IF components (dental, oral and peristomial plates and teeth), the present first author concluded that the similarities between the interbrachial frames of Matsumoto’s lines 1 and 2 were too great to be due to convergent evolution and that these taxa (Amphiuridae, Ophiotrichidae, Ophiocomidae and Ophionereidinae) constituted a previously unrecognised clade [25]. This clade was endorsed by Smith et al. [23] whose cladistic analysis combined molecular and morphological data (43 morphological characters, of which 40 were endoskeletal and 7 (17%) of these IF features): their infraorder Gnathophiurina comprised the four taxa named above together with the family Amphilepididae (which had previously been reduced to the rank of a subfamily within the Amphiuridae [26]). However, this has not been supported by more recent analyses involving both morphological [6] and phylogenomic [2,8,10] methodologies. Although the topology of their phylogenetic trees differs from that of Matsumoto [21], these analyses also place the Ophiocomidae (*sensu stricto*), Amphiuridae + Ophiactidae (*sensu stricto*) + Ophiotrichidae and *Ophioceramis/Ophioplocus januarii* (one of the 576 species in the dataset of O’Hara et al. [2]) in different clades and reinforce the view that the complex of traits peculiar to the IFs of these taxa has evolved independently on at least three occasions (Fig 3B). Indeed Thuy and Stöhr suggested [6] that similarities between the dental and oral plates of representatives of the Ophiocomidae and Amphiuridae + Ophiactidae + Ophiotrichidae might reflect “a yet unknown lifestyle-related independent acquisition”.

As noted by Matsumoto [21], these four families also show similarities in the morphology of their teeth and peristomial plates, structures that were not included in Thuy and Stöhr’s analysis [6]. The present investigation was prompted by the chance observation that, as well as showing long-known inter-taxon differences in relative size and fragmentation, peristomial plates also vary in their orientation with respect to the underlying oral plates, a feature that appears not to have been previously commented upon. A cross-class survey of the peristomial plates was then conducted with the aim of determining if the distribution of traits thus revealed, combined with available information on other IF characters, strengthened or weakened the apparent incongruence between IF comparative morphology and the latest phylogenetic trees.

## Materials and methods

The peristomial plates (PEPs) of 48 species (binomials in accordance with Stöhr et al. [27] and O’Hara et al. [2,9]) representing 21 of the 33 families delineated by O’Hara et al. [9] were examined (S1 Table). The specimens were either collected by the authors in the field or gifted from other sources (see ‘Acknowledgments’). Information on geographical locations is provided in S1 Table. None of the species is endangered or protected and, due to the absence of legal restrictions, no specific permissions were required for their collection. Most specimens were preserved by desiccation alone and the rest by desiccation following formalin fixation or by immersion in 70% or 96% ethanol. Generally one adult specimen of each species was selected, the main exception being *Ophiocomina nigra* (Abildgaard in O.F. Müller, 1789), nine specimens of which were used to assess intraspecific variability (see below). After the disc diameter was measured, the aboral portions of the disc and stomach were removed and the aboral side of the IF was exposed by digesting soft tissues gradually with bleach (NaOCl solution).

Observations were made with regard to three PEP characters: fragmentation, orientation and relative size. Each PEP consists of a single plate or two or more plates joined at mobile or rigid articulations. Most PEPs, whether single or fragmented, have an overall flattened shape and their orientation with respect to the oral plates is either *horizontal*, i.e. the PEPs form a roof over the neural groove on the doral side of the oral plates, or *inclined*, i.e. they form a vertical, or steeply sloping, partition slotted into the neural groove. The maximum width and maximum length of the PEPs were relativised against the maximum transverse width of the aboral half of the dental plate (DEP), which can be observed when viewing the aboral side of the intact interbrachial frame or the proximal side of a detached DEP (Fig 2A). This parameter was chosen because it enabled comparative data to be obtained from the widest range of previously published illustrations (see below). PEP and DEP parameters were measured using an eyepiece graticule in a Leica Wild M3Z stereomicroscope (Leica Microsystems GmbH, Wetzlar, Germany) usually at magnification ×40 or, in the case of very small specimens, in an Olympus CX40 compound microscope (Olympus UK Ltd., Southend-on-Sea, UK) with incident light and at a magnification of ×100. Preparations were photographed with a ToupCam S3CMOS05000KPA digital eyepiece camera (ToupTek Photonics, Hangzhou, China). Measurements were taken from one to five PEPs and corresponding DEPs per individual. In the case of *O*. *nigra*, the relative width and length of four or five PEPs of each of nine animals were estimated in this way in order to assess the size-dependent and size-independent variability of these values: four of the animals had a disc diameter of 11.0 mm and the others of 3.5, 5.0, 8.0, 14.0 and 17.0 mm respectively.

Data on representatives of an additional 8 families and on more species from the former 21 families were obtained from textual descriptions and images showing the aboral side of interbrachial frames in the published literature, particularly those of Lyman [16] and Matsumoto [21].

To illustrate the main types of interbrachial frame and their individual components, specimens of *Ophiacantha vivipara* Ljungman, 1871, *Ophiura lymani* **(**Ljungman, 1871), *Amphiura eugeniae* Ljungman, 1867 and *Ophioplocus januarii* were digested partly or wholly in bleach, rinsed in distilled water then ethanol, air dried at room temperature and mounted on aluminium stubs. After sputter-coating they were observed and photographed in a Philips XL-30 scanning electron microscope at the Museo Argentino de Ciencias Naturales “Bernardino Rivadavia”.

## Results

**Fig 4:**
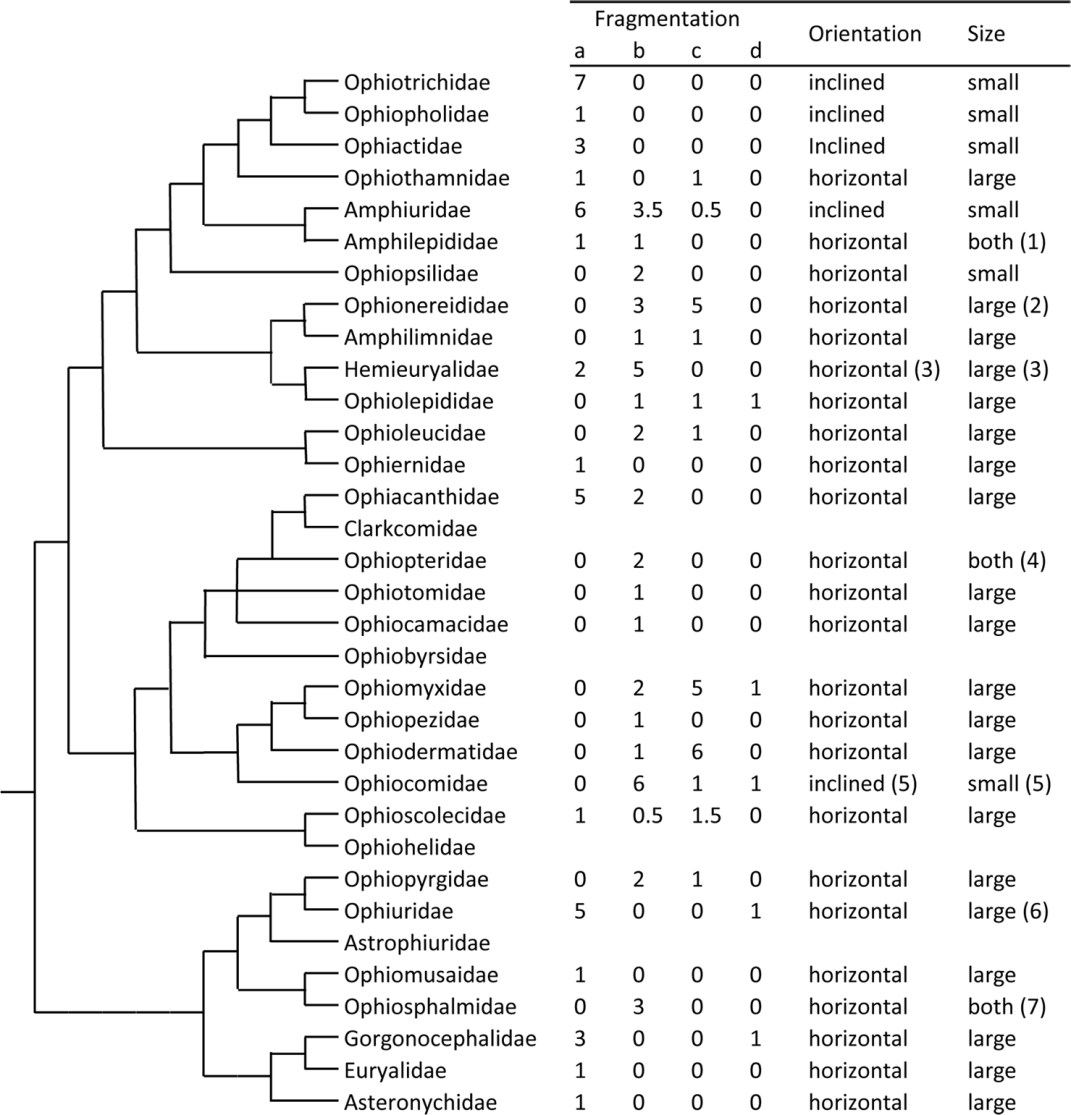
Character states of peristomial plates (PEPs): Summary of results. The cladogram is derived from O’Hara et al. [9] (slightly modified). Regarding fragmentation state, a = single, b = two parts, c = three parts, d = other; the values are the numbers of species showing each state; where a species shows two states, each state is assigned a value of 0.5. The numerals in parentheses refer to the following comments: (1) One species has small PEPs and one has large PEPs. (2) One species has small PEPs. (3) One species (*Ophioplocus januarii*) has inclined PEPs and two species (*O*. *januarii* and *Sigsbeia murrhina*) have small PEPs. (4) One species has small PEPs and one has large PEPs. (5) One species (*Ophiocomella ophiactoides*) has large horizontal PEPs. (6) One species (*Ophiocten sericeum*) has small PEPs. (7) One species (*Ophiolipus agassizii*) has small PEPs.

S2 Table shows the PEP character states of the 48 species from 21 families that were examined directly and S3 Table those of 14 species from an additional 8 families obtained from the published literature. S4 Table provides the PEP character states of 47 additional species from the former 21 families derived from the literature. The data are summarised in Fig 4.

### Fragmentation

**Fig 5:**
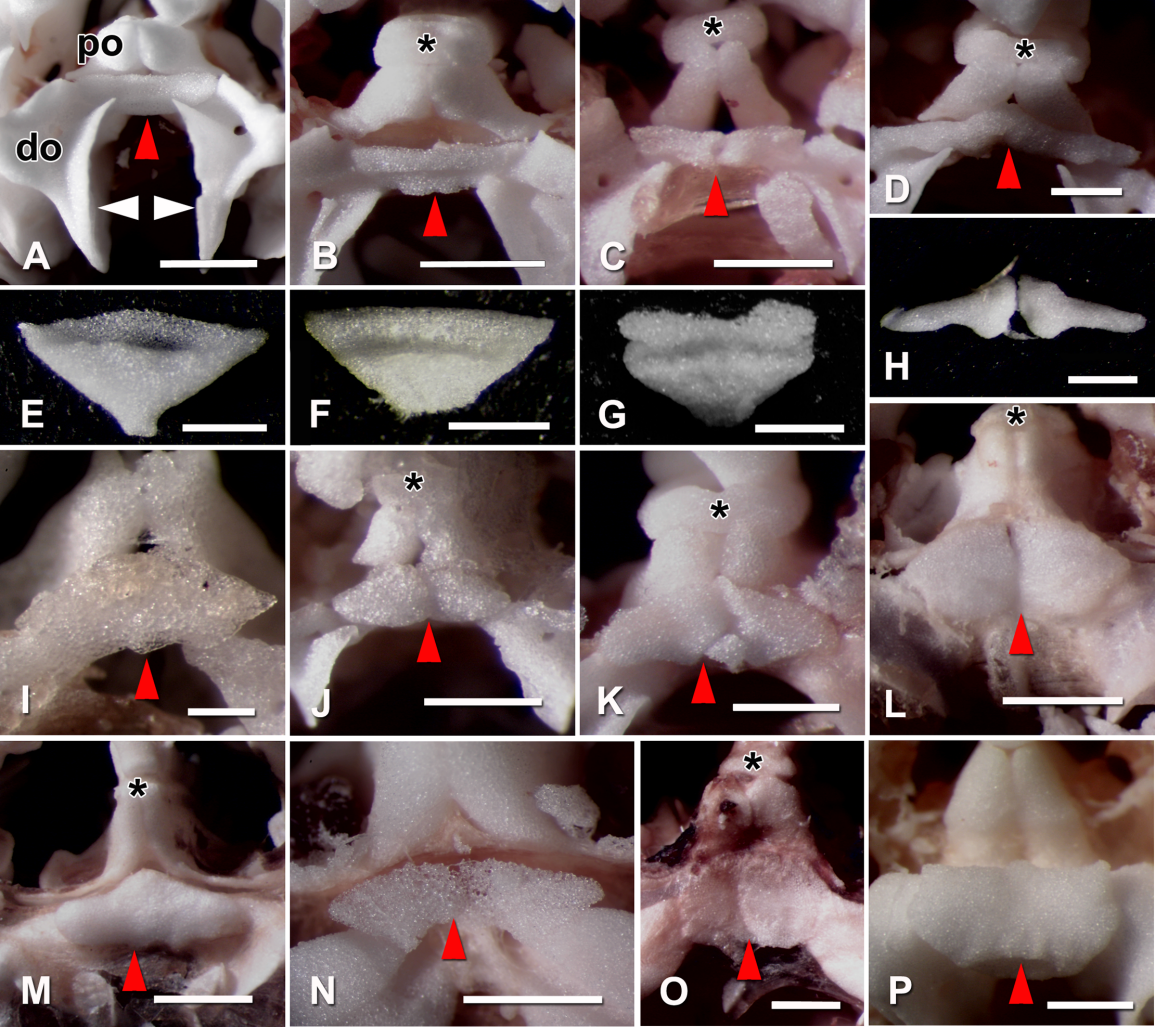
Light micrographs of peristomial plates *in situ* and detached. (A-D, I-P) Aboral views of single interbrachial frame units (“jaws”) of partly digested specimens. (E-H) Detached peristomial plates: distal side, aboral end at top. (A, E) *Ophiothrix fragilis* (Ophiotrichidae); (B, F) *Ophiopholis aculeata* (Ophiopholidae); (C, G) *Amphiura filiformis* (Amphiuridae); (D, H) *Ophiocoma pumila* (Ophiocomidae); (I) *Amphilepis ingolfiana* (Amphilepididae); (J) *Ophiopsila aranea* (Ophiopsilidae); (K) *Ophionereis reticulata* (Ophionereididae); (L) *Ophioplocus esmarki* (Hemieuryalidae); (M) *Ophiocomina nigra* (Ophiotomidae); (N) *Ophiomusa lymani* (Ophiomusaidae); (O) *Ophiura ophiura* (Ophiuridae); (P) *Astrophyton muricatum* (Gorgonocephalidae). In B and C the proximally projecting edges of the abradial muscle attachment areas (which are present in A) have been removed to enhance visualisation of the peristomial plates. do, distal part of oral plate; po, proximal part of oral plate; asterisk, dental plate; red arrowhead, peristomial plate; white arrowhead, abradial muscle attachment area. Scalebars: 1 mm (A, B, L, M, O); 0.5 mm (D-F, H, J, K, N, P); 0.4 mm (C); 0.25 mm (G, I).

In 39 (36%) of the 109 species for which information was obtained, each PEP in adult individuals was a single undivided plate; in 41 species (38%) each PEP consisted of only two lateral halves adjoined at their abradial edges; in 24 species (22%) the PEPs resembled the latter but had an additional unpaired plate, usually at the distal edge; and the PEPs of the remaining five species either resembled the latter two types in comprising two main plates but had two or more additional small plates (three species), or they consisted of three or more imbricating plates (one species) or of a large unpaired plate with smaller plates at its distal edge (one species) (Fig 5). The fragmentation state showed little consistency at any taxonomic level. Amongst suprafamilial clades the only exception to this was the notable prevalence of single PEPs in the suborder Gnathophiurina (the branch comprising the top six families in Fig 4). Regarding within-family variability, of the 15 families represented by two or more genera, only two (Ophiotrichidae and Ophiosphalmidae) had a consistent fragmentation state. The fragmentation state may be more stable within genera: 12 of the 24 genera represented by two or more species showed interspecific uniformity and 12 interspecific variability. There was, however, evidence for plasticity within species and within individuals. For example, although Matsumoto [21] described and illustrated *Amphipholis kochii* as having single PEPs, the specimen examined by the present authors had unambiguously two-part PEPs. Matsumoto [21] illustrated variation within individuals of *Amphiura tachydisca* and *Ophiohyalus gotoi*, which the present authors also observed in *Ophiocomina nigra*: of the nine individuals that were examined, two (both with disc diameter 11 mm) had four two-part PEPs (the usual condition) and one three-part PEP.

### Relative size

**Fig 6:**
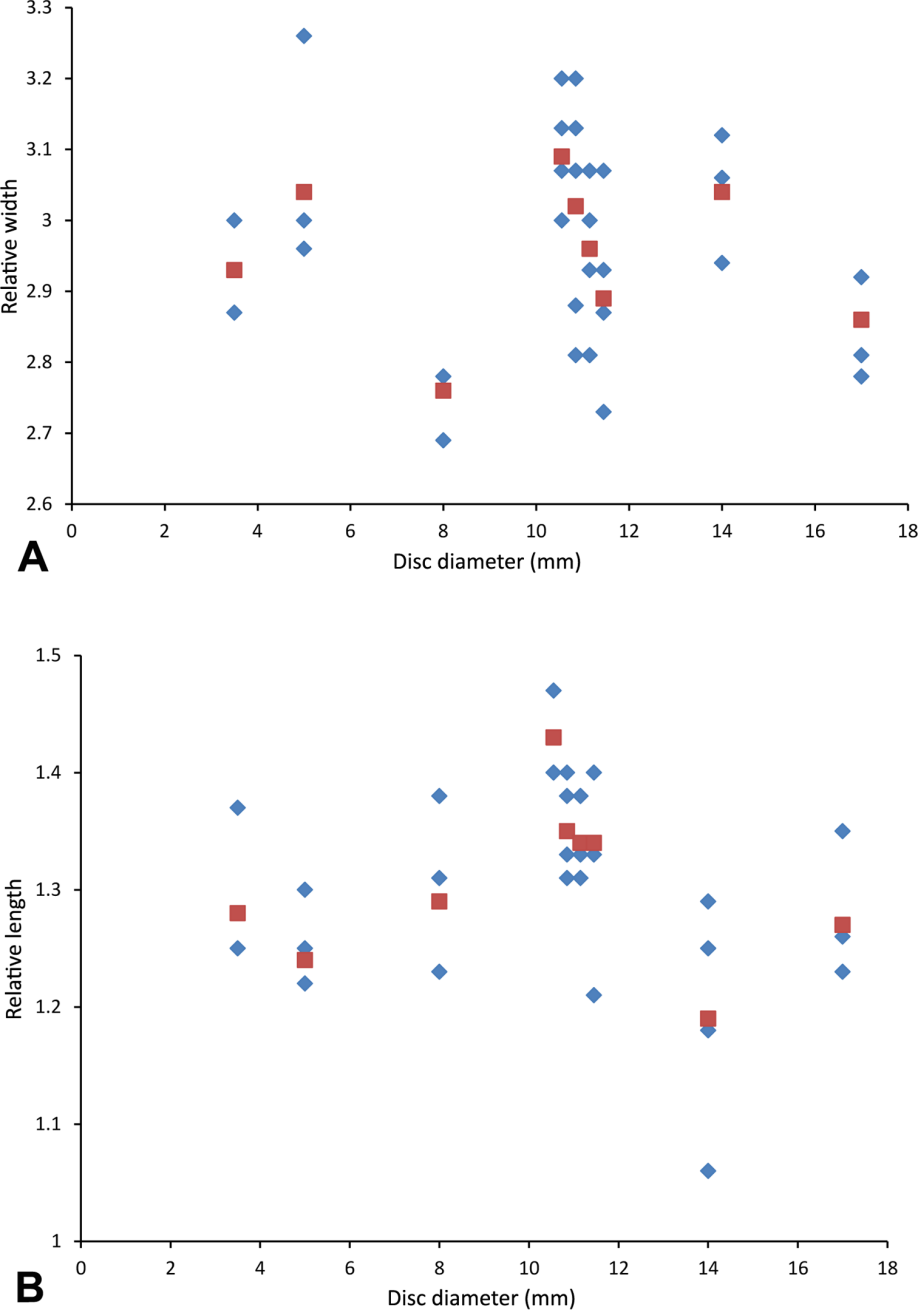
**Peristomial plates of *Ophiocomina nigra*: relationship between (A) disc diameter and relative width and (B) disc diameter and relative length.** Results from nine animals, four with disc diameter 11 mm and the others with disc diameters 3.5, 5.0, 8.0, 14.0 and 17.0 mm respectively. The relative dimensions of four or five PEPs of each animal were estimated; blue diamonds indicate individual values and brown squares indicate means for each animal; the values for the four animals with disc diameter 11 mm are staggered slightly along the x-axis. Pearson product-moment correlation indicates no significant association between disc diameter and relative width (r = 0.0016, d.f. 41, P > 0.05) or relative length (r = 0.0328, d.f. 41, P > 0.05).

In view of the generally small sample sizes from which the relative width and length of the PEPs were calculated, *Ophiocomina nigra* was used as an indicator of within-species variability. Fig 6 shows that within-individual variability in relative dimensions was about as great as between-individual variability and, for animals with the same body size (i.e. disc diameter), there was considerable between-individual variability in the range of values and mean values. There was no evidence for an association between body size and relative dimensions over a disc diameter range of 3.5–17.0 mm (Fig 6). This provides some reassurance that the relative dimensions of one PEP from one adult individual may be representative of the species.

**Fig 7:**
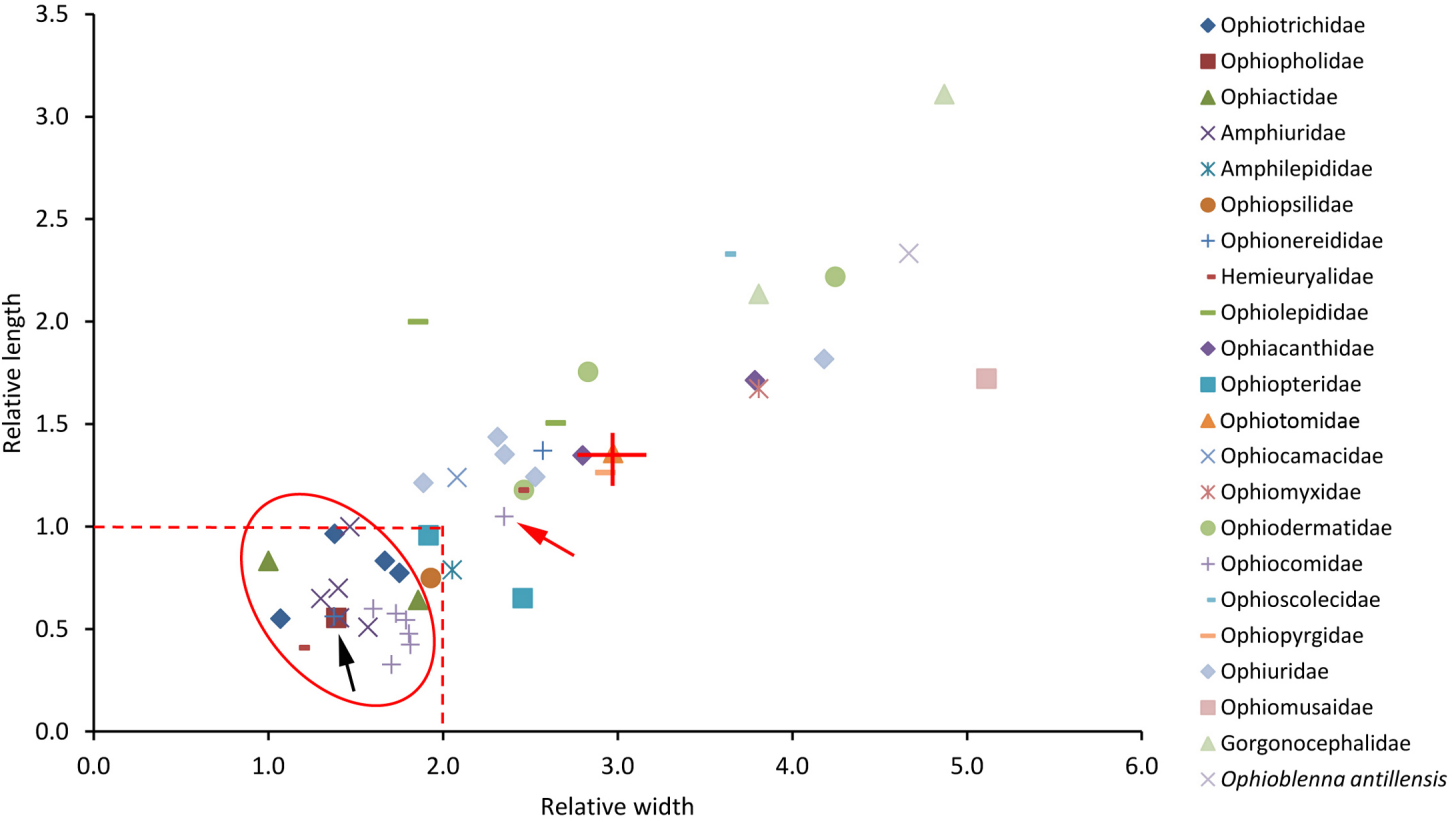
Relative dimensions of the peristomial plates of directly examined species. Each symbol represents one species. *Ophioblenna antillensis* (formerly placed in the Ophiacanthidae) is currently *incertae sedis*. The broken lines are the upper limits of the relative dimensions of ‘small’ PEPs as defined herein. The red oval encloses all species with inclined PEPs (and a single species with horizontal PEPs–*Ophionereis reticulata*). The black arrow indicates a cluster of three species–*Acrocnida brachiata*, *Ophionereis reticulata* and *Ophiopholis aculeata*) and the red arrow indicates *Ophiocomella ophiactoides* (the only ophiocomid that does not have small inclined PEPs). The red bars superimposed on the symbol for *Ophiocomina nigra* show the ranges of values obtained from nine individuals of this species (disc diameters 3.5–17.0 mm).

Fig 7 is a plot of relative length against relative width of PEPs from the directly observed species. This confirms Matsumoto’s observation that the PEPs of species belonging to the families (all *sensu* Matsumoto [21]) Ophiotrichidae, Amphiuridae (i.e. current Amphiuridae + Ophiactidae + Ophiopholidae) and Ophiocomidae (i.e. current Ophiocomidae + Ophiopsilidae + Ophiopteridae) are small in comparison with those of species belonging to other families: all had a relative width (RW) of 2 or less and a relative length (RL) of 1 or less, with the exception of one of the two *Ophiopteris* species and the ophiocomid *Ophiocomella ophiactoides*. It is notable that the ophiocomids (apart from *O*. *ophiactoides*) form a tight cluster characterised by their particularly low relative length. In view of the intraspecific variability demonstrated by *Ophiocomina nigra* (Fig 6), one other species—*Amphilepis ingolfiana* (family Amphilepididae)—should be regarded as having small PEPs (i.e. RW≤2 + RL≤1), which does not accord with Matsumoto’s view [21] that the Amphilepididae had “very large” PEPs (based, however, on one species–“*Amphiactis umbonata*”–subsequently renamed *Histampica umbonata* and reassigned to family Ophiothamnidae [2,9,26]).

**Fig 8:**
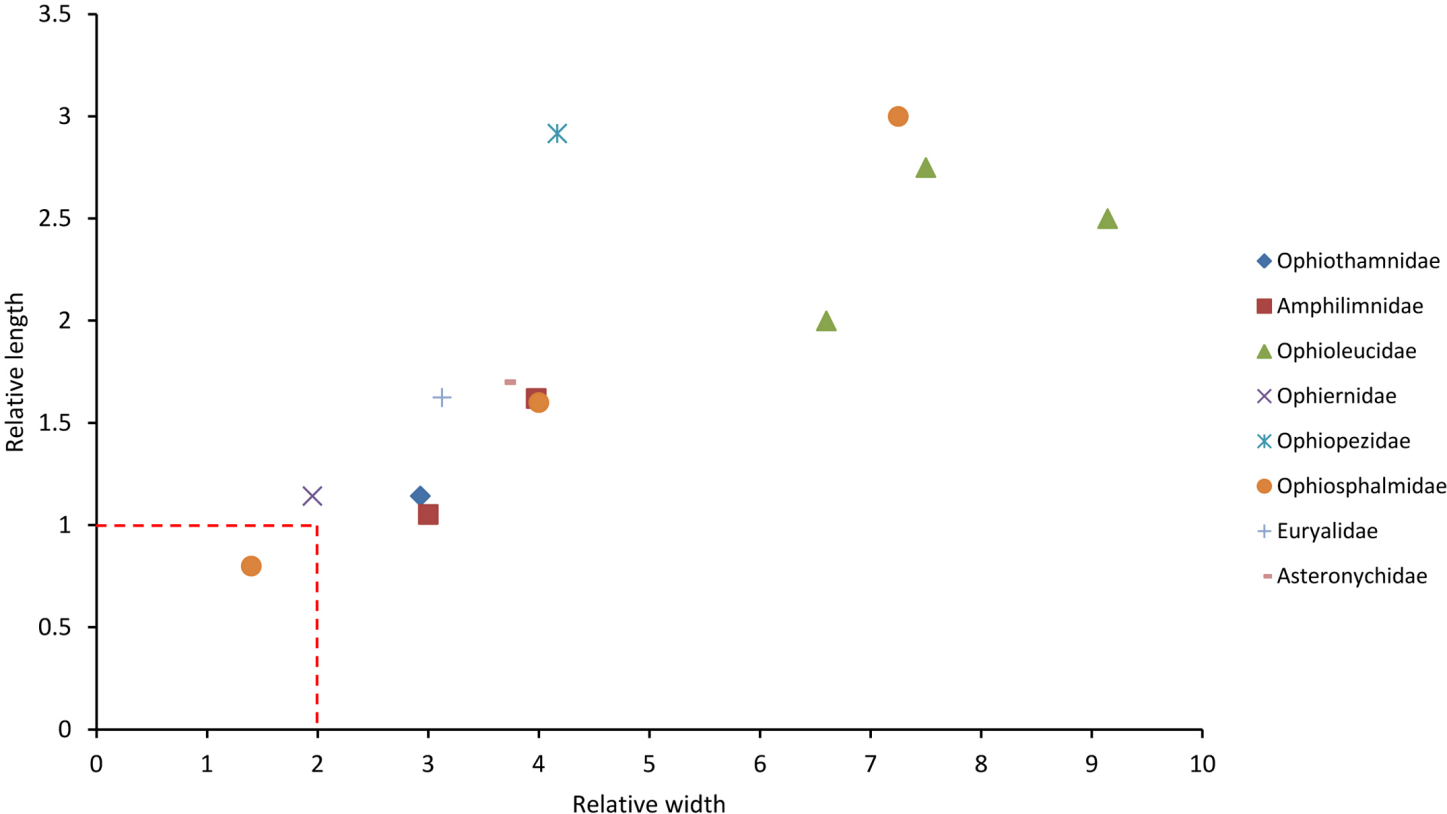
Relative dimensions of the peristomial plates of species from families not represented in Fig 7 (data obtained from the published literature). The broken lines are the upper limits of the relative dimensions of ‘small’ PEPs as defined herein.

**Fig 9:**
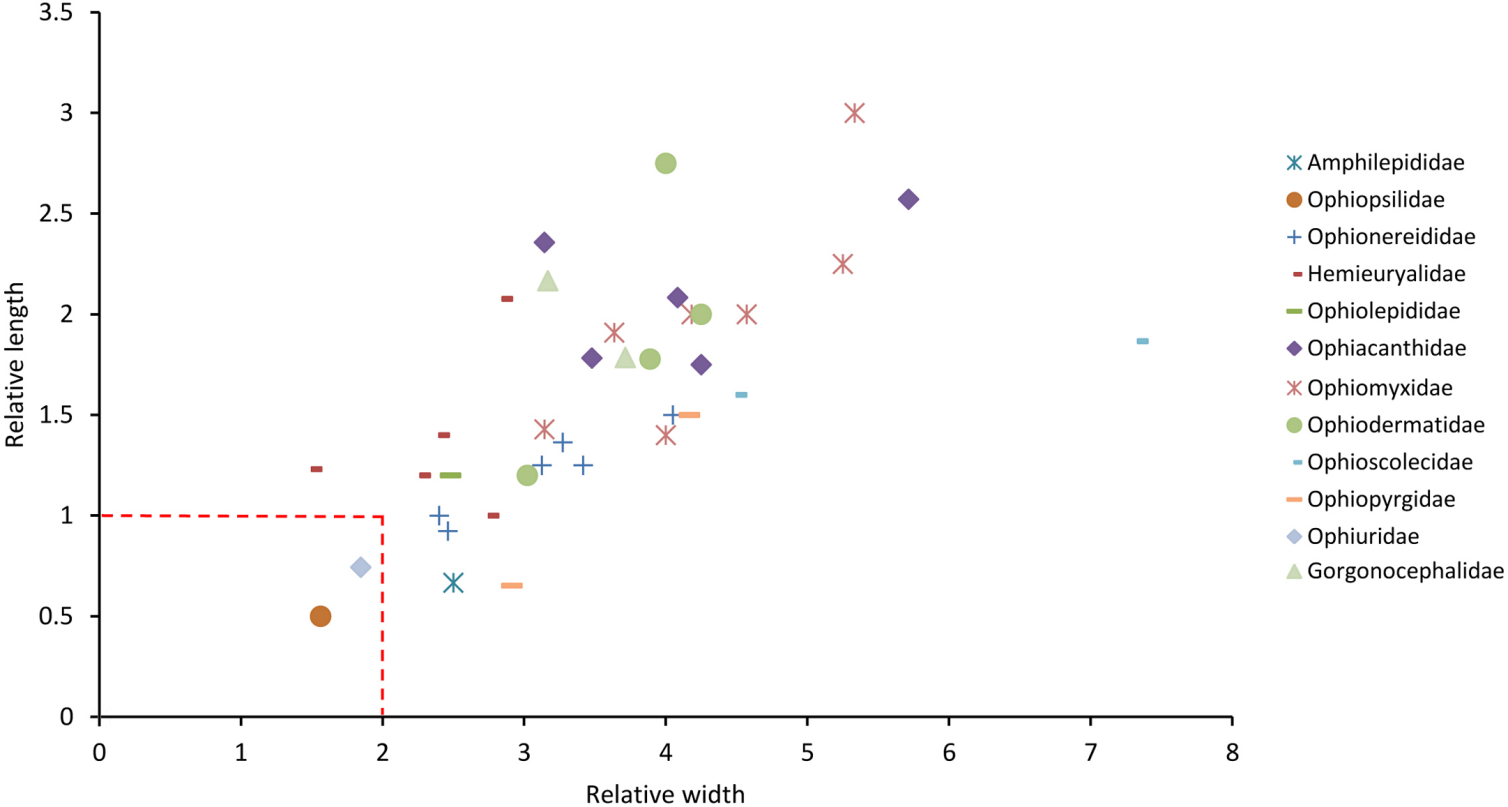
Relative dimensions of the peristomial plates of additional species from families represented in Fig 7 (data obtained from the published literature). The broken lines are the upper limits of the relative dimensions of ‘small’ PEPs as defined herein.

Data on representatives of an additional eight families obtained from the literature suggested that none had ‘small’ PEPs (as defined above) except for *Ophiolipus agassizii*, one of three representatives of the Ophiosphalmidae (Fig 8). Data extracted from the literature on an additional 47 species belonging to 16 of the 21 directly observed families are incomplete, because it was not possible to measure the length, and therefore calculate the relative length, of inclined PEPs from the illustrations of any species of Ophiotrichidae, Ophiactidae, Amphiuridae and Ophiocomidae (S4 Table). However, the PEPs of all representatives of these families had a small relative width and, with the exception of *Sigsbeia murrhina* (Hemieuryalidae) and *Ophiocten sericeum* (Ophiuridae), none of the 37 species belonging to families other than these had small PEPs (RW≤2 + RL≤1) (Fig 9).

The validity of using published drawings, especially those of Lyman [16] and Matsumoto [21], to estimate PEP relative size depends on the faithfulness of the images. Matsumoto [21] illustrated the IFs of six of the species that were examined directly in the present investigation. To check his accuracy, relative dimensions were calculated from his illustrations of these six species (RW only of *Ophiopholis aculeata*, *Amphipholis kochii*, *Ophionereis reticulata* and *Ophioplocus januarii*; RW and RL of *Ophiacantha bidentata* and *Ophiolepis impressa*). For all six species the values fell within the same size categories (i.e. ‘small’ or ‘large’) as did those derived from directly examined specimens. Lyman [16] did not illustrate the PEPs of any of the directly examined species.

### Orientation

Of the 109 species for which data were obtained, 80 had horizontal PEPs forming a roof over the neural groove on the dorsal side of the oral plates, 27 had inclined PEPs slotted into the neural groove, and the PEP orientation of two species was unclear (S2–S4 Tables). Inclined PEPs were present in all species of Ophiotrichidae (apart from one species with uncertain orientation), Ophiactidae (apart from one species with uncertain orientation), Amphiuridae and Ophiocomidae (apart from *Ophiocomella ophiactoides*) (Fig 5A–5D) and in only one species from any other family–*Ophioplocus januarii* (Hemieuryalidae) (Figs 1Da and 2B). All inclined PEPs without exception were small (RW≤2 + RL≤1), although not all small PEPs were inclined (Fig 7). The inclined PEPs of all directly examined species of Ophiotrichidae and Ophiactidae and all but one species of Amphiuridae were single and roughly the shape of an obtuse isosceles triangle with the apex pointing orally and with a transverse groove (in which the circumoral water vascular ring is located *in vivo*) on the distal side near the aboral edge (Fig 5E–5G); the inclined PEPs of all directly examined species of Ophiocomidae and one amphiurid (*Amphipholis kochii*) consisted of two parts (Fig 5D and 5H).

## Discussion

### Peristomial plate morphology and its implications

The peristomial plates (PEPs) were at one time thought to be derived, like most other components of the interbrachial frame (IF), from segmental ossicles (the first ambulacrals) of the internal arm skeleton [17]. However, the skeletal ontogenesis of the IF (at least that of two amphiurid species) suggests that this is incorrect and that the PEPs are secondary structures [28,29], which probably evolved in response to a specific functional demand, such as mechanical stabilisation of the IF or the physiological need to isolate the underlying circumoral nerve ring in a separate compartment.

Lyman [16] appears to be the first to have included information on PEPs (mainly size and fragmentation state) in taxonomic descriptions: PEPs are mentioned in 48 of his 85 generic diagnoses. Lyman made no reference to PEPs in his ten articles on ophiuroid systematics that preceded his 1882 magnum opus [16] nor in the single paper that followed it (for a list of his publications, see [30]). With the notable exception of Matsumoto’s inclusion of PEP features in his diagnoses of families and orders [20,21], PEPs (and other internal IF characters) largely dropped from view in the subsequent taxonomic literature, possibly because they cannot be observed directly without destructive dissection [7,31]. Amongst the rare exceptions are a comment by Duncan [32] on differences between PEP traits of two “*Ophiophragmus*” species (both subsequently assigned to other genera), the mention by Verrill [33] of PEPs in (only) two of his 12 familial diagnoses, the observation by Madsen [34] that the PEP fragmentation state varies between genera of Ophioleucinae, and the use by Smith et al. [23] of PEP morphology in their cladistic analysis.

The present investigation demonstrated that, whilst PEP fragmentation state shows little consistency at any taxonomic level, relative size segregates a group of families comprising species that consistently have small PEPs (‘small’ being defined herein as RW≤2 + RL≤1), viz. Ophiotrichidae, Ophiopholidae, Ophiactidae, Amphiuridae, Ophiopsilidae and Ophiocomidae (all *sensu* O’Hara et al. [2]), thus confirming quantitatively Matsumoto’s remarks on the small size of the PEPs in these clades [21]. On the basis of the range of species for which there are data, it appears that the majority of other families have consistently large PEPs, the few heterogeneous families being: Amphilepididae with one small-PEP species (SP) and one large-PEP species (LP) included in the present survey, Ophiopteridae (1SP + 1LP), Ophionereididae (1SP + 7LP), Ophiosphalmidae (1SP + 2LP), Hemieuryalidae (1SP + 5LP) and Ophiuridae (1SP + 5LP).

This enquiry identified another trait shared almost exclusively by those families with small PEPs (apart from the Ophiopsilidae): their PEPs are inclined and slotted into the neural groove of the oral plates, in contrast to the horizontal PEPs that form a roof over the neural groove in all other ophiuroid families. Only one species from any other family–the hemieuryalid *Ophioplocus januarii–*has this feature. PEP inclination appears not to have been previously reported. In his descriptions of most genera now assigned to these families (viz. *Ophiothrix*, *Amphiura* and *Ophiactis*) and of *O*. *januarii*, Lyman [16] asserted that PEPs were either absent or present as a “thin crust”, and for *Ophiocoma* he noted that “The nerve ring is scarcely covered by the linear, narrow, peristomial plate…”, although all the *Ophiocoma* species (and other ophiocomids) examined in the present investigation have robust PEPs that completely cover the nerve ring (Fig 5D). Matsumoto [21] clearly illustrated the inclined orientation of the PEPs in several species belonging to these families (including *Ophiothrix nereidina*, *Ophiopholis aculeata*, *Amphipholis kochii* and *Ophiarthrum elegans*) and in *O*. *januarii*, but made no reference to this trait in his main text or in the detailed ‘General Explanation of Plates’.

**Fig 10:**
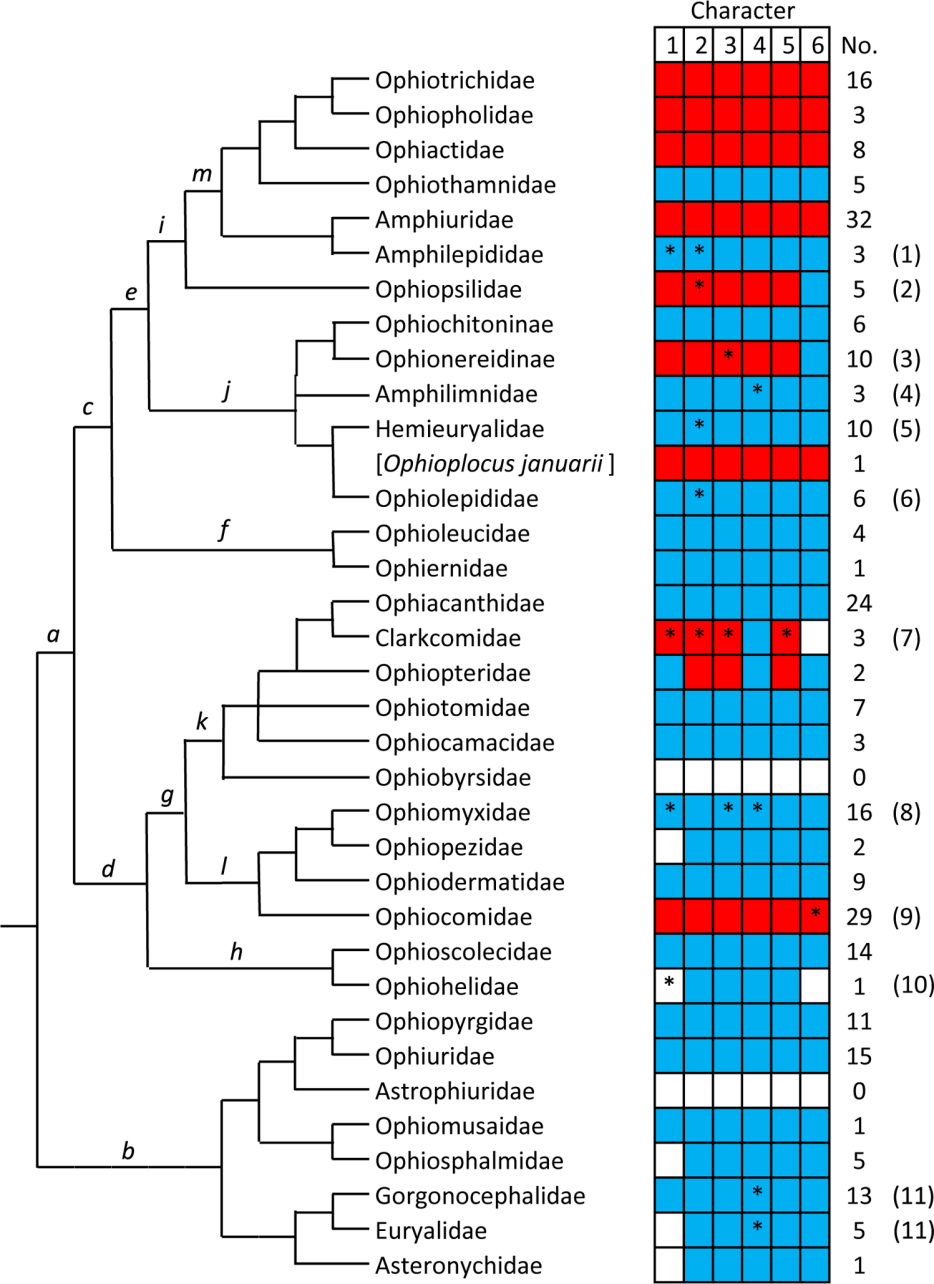
Familial distribution of IF character states, based on information accumulated to date (S5 Table). The cladogram is derived from O’Hara et al. [9] (modified by the separation of *Ophioplocus januarii* from other hemieuryalids and the representation of family Ophionereididae as subfamilies Ophionereidinae and Ophiochitoninae). Characters are numbered as in Table 1. Blue denotes type A and red type B character states. White denotes lack of data. The numbers of species for which information is available are also given. Lower case italicised letters indicate clades to which reference is made in the text. Asterisks and numerals in parentheses refer to the following comments: (1) The dental plates of two species are type A and that of the third (*Amphilepis ingolfiana*) has a B-like aboral perforation. The teeth of this last species also have an imperforate median cusp (apparently also present in certain other *Amphilepis* species [26,45]), which may be a vestige of the imperforate cap present in most other families of clade *m* (suborder Gnathophiurina). (2) There is information on the abradial muscle attachment area of four species, three of which are type B and one type A. (3) There is information on the abradial muscle attachment area of five species, four of which are type B and one type A. (4) The adradial muscle attachment area approaches the type B state, but lacks a pronounced aboral expansion and narrow oral region. (5) Most hemieuryalids have type A dental plates. However, *Ophioplocus januarii* and four *Ophiozonella* species have type B dental plates. (6) Although most ophiolepidids have type A dental plates, one species has an “imperfect” perforation and another has distinctly type B perforations. (7) Of the five characters for which there is information, only the type A adradial muscle attachment area is consistent across the three species. All other characters are type A in one species (*Clarkcoma australis*) and type B in the other two (*C*. *bollonsi* and *C*. *canaliculata*). (8) The IF traits of the Ophiomyxidae are consistently type A, with the exception of the teeth of the six *Ophioconis* species for which information was obtained. These have broad teeth with a cap of imperforate stereom, which in most (but not all) species has a denticulate edge. The latter feature suggests that these have evolved from *Ophiomyxa*-like teeth, which have a proximal edge of imperforate spikes ([36]; Wilkie pers. obs.). The abradial and adradial muscle attachment areas of *Ophioconis cincta* and *O*. *cupida* also approach the B condition. (9) The character states of all ophiocomids are consistently type B, with the exception of *Ophiocomella ophiactoides*, which has type A peristomial plates. (10) Some ophiohelids have broad, though flattened, teeth, those of *Ophiothauma heptactis* also having an imperforate proximal edge [46]. (11) The adradial muscle attachment area of a few gorgonocephalid and euryalid species approaches the type B spoon-with-narrow-handle shape.

Recently proposed phylogenetic trees [2,6,9] (Fig 3B) imply that small size and inclined orientation are derived PEP conditions and that small inclined PEPs have evolved independently in at least three clades: suborder Gnathophiurina (clade *m* in Fig 10; it will be argued below that the PEP morphology of the Ophiothamnidae and Amphilepididae has reverted to the ancestral state), family Ophiocomidae and *Ophioplocus januarii*. Viewed in isolation, this convergence of PEP traits would be unremarkable, since the developmental mechanisms controlling PEP size and orientation are likely to be relatively simple and therefore compliant to evolutionary forces. However, the fact that this is just one of a range of similarities between the IFs of these same three clades indicates that something more challenging for ophiuroid systematics is afoot.

### Other IF characters and their implications

The present investigation is part of a wider survey of IF morphology being conducted by the authors, which so far has accumulated information on over 270 ophiuroid species (obtained by direct observation and from the published literature: S5 Table). This indicates that the distribution of PEP traits discussed above is strongly correlated with that of several other IF character states. The latter, which pertain to the teeth, dental plates, oral plates and associated soft tissue components, are explicated in Table 1 and the distribution of all IF character states is shown in Fig 10. Since the latter is based on data from around 10% of extant ophiuroid species, it must be regarded as ‘work in progress’ and any inferences derived from it can be only provisional at this stage. On the basis of available information it appears that combinations of IF character states are quite stable at the family level, i.e. each family consists of species all or most of which share the same trait combination. A major exception to this is the Ophionereididae (*sensu* O’Hara et al. [2,9]) in which there is a consistent distinction between *Ophionereis*, all of whose attributes are type B apart from their PEPs, and the other genera (*Ophiochiton*, *Ophiodoris* and *Ophioplax*) whose traits are uniformly type A. *Ophionereis* constitutes the former subfamily Ophionereidinae and the other genera were formerly assigned to the subfamily Ophiochitoninae, these subfamilies having been abandoned because phylogenomic analysis suggested that Ophiochitoninae was paraphyletic with respect to *Ophionereis* [2,9,35]. Heterogeneity is also evident in the Hemieuryalidae, which exhibits predominantly type A traits, but includes *Ophiozonella* species with type B dental plates and *Ophioplocus januarii*, which, in contrast to other *Ophioplocus* species and other hemieuryalids, has the full complement of type B character states.

**Table 1:** Type A and B character states.

Character	Type A (Figs 1A, 1B and 2A)	Type B (Figs 1C, 1D and 2B)
1	**Teeth**	
	Elongate spiniform to broad; lacking a cap of imperforate or denser fenestrated stereom.	Broad, blunt- or square-tipped; having a cap of imperforate or denser fenestrated stereom. (1)
2	**Dental plate**	
	Entire or divided; all tooth sockets in the form of depressions that do not continue as wide perforations through the plate.	Entire; at least some aboral tooth sockets in the form of wide perforations that are divided into two lateral halves by a complete or incomplete vertical bar of stereom. (2)
3	**Oral plate: abradial muscle attachment area**	
	Small; i.e. its profile is contained within the lateral profile of the whole plate; the attachment area is not interrupted by microstructurally differentiated stereom.	Large and located on a (human) ear-shaped flange that projects beyond the lateral profile of the whole plate; the attachment area usually has branching or radiating interruptions of microstructurally differentiated stereom. (3)
4	**Oral plate: adradial muscle attachment area**	
	Oral-dominant, i.e. always present on, and usually restricted to, the oral half of the adradial surface; if it extends more aborally, the oral portion is not shaped like a narrow spoon ‘handle’.	Aboral-dominant, i.e. always present on the aboral half of the adradial surface, the aboral portion usually expanding like the bowl of a spoon and the oral portion either absent or having the shape of a narrow ‘handle’. (4)
5	**Oral plate: adradial articular area**	
	Adradial outline usually reniform; the proximal edge is convex and the distal edge notched with distally projecting oral and aboral convexities.	Adradial outline consists of a distally projecting oral convexity and an aboral portion in the form of a straight vertical edge. (5)
6	**Peristomial plate**	
	Relatively large; orientated horizontally, i.e. forming a roof over the neural groove.	Relatively small; orientated vertically, or nearly so, within the neural groove. (6)

The numbers in parentheses refer to the following comments: (1) The microstructure of type A and B tooth types has been described and illustrated with scanning electron micrographs by Medeiros-Bergen [36–38]. Most examined type B teeth have a cap consisting of imperforate stereom (Fig 1Cc), which has been described variously in the literature as “enamelled”, “hyaline” or “glassy”; a cap consisting of more densely fenestrated stereom has been observed in *Ophiopholis aculeata* (Ophiopholidae), *Ophiactis asperula* (Ophiactidae), *Acrocnida brachiata*, *Amphiura chiajei* (both Amphiuridae) and *Ophioplocus januarii* (Hemieuryalidae) ([37,39]; Wilkie pers. obs.) (Fig 1Dc). The cap of type B teeth may provide greater resistance to abrasive damage resulting from the close apposition of the teeth when the IF is maximally constricted. (2) In type B dental plates (Fig 1Cb and 1Db) each perforation houses a pair of large dental muscles, which at one end are attached to the base of the tooth and at the other to the proximal edge of the oral plates. In IFs with type A dental plates, the dental muscles are attached to the tooth base and to the dental plate. (3) This is the attachment area for the external interradial muscle (Fig 2A and 2B), which has a much larger cross-sectional area in type B than in type A oral plates (Fig 1Ae, 1Be, 1Ce and 1De). The interruptions in the attachment area consist of ridges or grooves that branch or radiate from the distal edge of the attachment area and consist of coarser stereom with wider fenestrations than the microgalleried stereom of the regions to which muscle fibres are attached (Fig 1Ce and 1De). They represent sub-areas to which muscle fibres are not attached due to the presence within the muscle of diverticula from the genital bursae, which enter the muscle at its distal side [19,40–43]. The physiological significance of the bursal diverticula is unclear: at its thinnest, the diverticular wall in both *Ophiothrix fragilis* (Ophiotrichidae) and *Ophiocomella ophiactoides* (Ophiocomidae) consists of a cuboidal luminal epithelium and a squamous visceral epithelium, suggesting that it is not adapted for gaseous exchange (Wilkie pers. obs.). Branching interruptions are characteristic of Amphiuridae, Ophiactidae and Ophiotrichidae, and radiating interruptions of Ophiocomidae, though the amphiurids *Ophiophragmus wurdemanii* and *Amphioplus iuxtus* and the ophiotrichid *Macrophiothrix longipeda* have ophiocomid-like radiating interruptions [16,24] and the ophiocomids *Ophiocomella ophiactoides*, *Ophiocomella* sp. and *Ophiocoma pusilla* have branching interruptions ([24,44]; Wilkie pers. obs.). A few species from these families lack interruptions, e.g. *Ophiothela danae* (Ophiotrichidae), *Ophiactis abyssicola* (Ophiactidae), *Amphipholis sobrina* (Amphiuridae). (4) This is the attachment area for the radial muscle (Fig 2B). Thuy & Stöhr [6] previously noted that the adradial muscle attachment areas of ophiotrichids, ophiopholids, ophiactids, amphiurids, ophiopsilids, ophiocomids and some ophionereidids (all *sensu* Thuy & Stöhr) feature an aboral expansion shaped like the bowl of a spoon, usually with a narrow oral ‘handle’ that can be continuous or interrupted (Fig 1Cd and 1Dd). (5) The straight vertical distal edge of type B adradial articular areas is the distal margin of a wide, parallel-sided vertical groove which is present on the distal side of the two oral plates that articulate at the adradial joint. As it faces the proximal side of the first vertebral ossicle of the arm, it is called the advertebral groove herein (Figs 1Cd, 1Da, 1Dd and 2B). The advertebral groove houses a prominent ligament (the distal radial ligament) whose fibres extend horizontally and transversely between the lateral walls of the groove (Wilkie pers. obs.). Both the groove and the strong development of the distal radial ligament are peculiar to type B IFs. (6) ‘Relatively large’ = (relative width > 2 and relative length > 1) and ‘relatively small’ = (relative width ≤ 2 and relative length ≤ 1).

There appears to be much less consistency in the distribution of IF traits at suprafamilial levels. According to the phylogeny of O’Hara et al. [2,9], extant ophiuroids comprise two superorders, which they named Ophintegrida and Euryophiurida (clades *a* and *b* in Fig 10). The Euryophiurida has, with very few exceptions, uniformly type A IFs, type B traits therefore being more or less restricted to the much larger Ophintegrida. The latter consists of two major clades (*c* and *d* in Fig 10; unnamed and unranked by O’Hara et al. [2,9]. Whilst type B character states are much more prevalent in clade *c* (five families having all, or all but one, B traits), they are also present in three families within two suborders (*k* and *l* in Fig 10) of clade *d*: the Ophiocomidae is consistently type B and there are different mixtures of A and B features in the Ophiopteridae and certain species of Clarkcomidae. However, even in clade *c* there is little correlation between IF morphology and suprafamilial taxonomy.

What is the explanation for the distribution of type B traits shown in Fig 10 and in particular what have been the respective contributions of convergence and phylogenetic affinity to this distribution? This is pertinent because there has been much speculation about the role of convergence in ophiuroid evolution [6–8], which harmonises with the ongoing debate about the relative importance and wider implications of convergence in the evolution of all organisms [47–50]. Matsumoto [21] inferred that the IFs of Ophiotrichidae, Amphiuridae and Ophiocomidae (*sensu lato*) were derived and the distribution of traits that characterise these IFs certainly supports that view: since type B character states are very rare in superorder Euryophiurida and occur only sporadically in superorder Ophintegrida (Fig 10), the alternative proposition–that type A character states are derived–would require there to have been a much greater and scarcely credible incidence of trait reversal. This is reinforced by the fossil record: whereas extant families originating in the Early Mesozoic (viz. Ophiolepididae, Ophiacanthidae, Ophiodermatidae and Ophiuridae) have type A IFs, those families with the full complement of type B character states (viz. Ophiotrichidae, Ophiactidae, Amphiuridae and Ophiocomidae) originated in the Late Mesozoic [3,51,52]. If the derived state of type B traits is assumed, it appears at first sight that they have evolved independently in at least eight lineages (Fig 10). However, closer examination of suborder Gnathophiurina (clade *m* in Fig 10) is instructive. This clade was first recognised (as order Gnathophiurida) by Matsumoto [20] who included all the sub-clades shown in Fig 10, except for the family Ophiothamnidae, which was added by O’Hara et al. [2] on the basis of phylogenomic affinity. Four of the six families comprising the Gnathophiurina have fully type B IFs and indeed Matsumoto’s diagnosis of this clade referred to the presence of small peristomial plates and oral plates “with well-developed lateral wings” (i.e. character state B3 in Table 1) [20]. The other two families of the Gnathophiurina–Ophiothamnidae and Amphilepididae, which belong to separate suprafamilial lineages, have fully type A IFs. Due to external skeletal features, *Ophiothamnus* Lyman, 1869, the type genus of the Ophiothamnidae, was previously placed in the family Ophiacanthidae [53]. However, it has phenotypic attributes that indicate its gnathophiurine relationship: (1) the articular tubercles on the lateral arm plates of *Ophiothamnus* species are of a type (consisting of two parallel ridges) shared with the other families of the suborder and with the Ophiopsilidae [4,5,53]; (2) *Ophiothamnus* species have the capacity for disc autotomy [53–55], an aptitude that is characteristic of gnathophiurine ophiuroids and of amphiurids in particular ([56]: Table XIII; [35]). It has been suggested that the fused muscle and nerve openings of the articular tubercles of *Ophiothamnus* lateral arm plates is a paedomorphic feature [4]. This observation and the fact that all three ophiothamnid genera (*Ophiothamnus*, *Ophioleila* A.H. Clark, 1949 and *Histampica* A.M. Clark 1970) consist entirely of small to very small species (maximum adult disc diameter 2–10 mm: sources mainly holotype descriptions referenced by Stöhr et al. [27]), is a strong indication that the ancestral ophiothamnid underwent a drastic paedomorphic process resulting in the loss of most gnathophiurine traits, including the reversion of all type B character states back to the type A condition. A similar argument applies to the Amphilepididae, both genera of which (*Amphilepis* Ljungman, 1867 and *Ophiomonas* Djakonov, 1952) also comprise small to very small species (maximum adult disc diameter 3–11 mm: sources as above). The Amphilepididae has been described as “a paedomorphic family with reduced characters” [9]. However, in this case the paedomorphic process appears to have resulted in less drastic trait reversal than in the Ophiothamnidae, since aspects of amphilepidid morphology have always suggested an affinity with the Amphiuridae (to the extent that amphilepidids were regarded as forming a subfamily of the Amphiuridae by Clark [26]), and some amphilepidids have retained vestigial type B IF traits (Fig 10). It is therefore possible that the type B IF is an apomorphy of the Gnathophiurina and that its absence from the Ophiothamnidae and Amphilepididae is due to paedomorphic trait reversal. This idea is predicated on the assumption that during ontogeny the type B condition is preceded by a type A stage. Whilst there is evidence that this is true of the teeth and peristomial plates of amphiurids [28,29,57], it needs to be confirmed for the dental and oral plates of the Amphiuridae and of other families with type B IFs.

The family Ophiopsilidae, which according to O’Hara et al. [9] is sister group to the Gnathophiurina within unnamed clade *i*, exhibits all type B IF character states, except for its large horizontal PEPs (Figs 5J and 10). Whether this type A feature is a plesiomorphy or another example of trait reversal may be clarified by consideration of the distribution of IF character states within clade *j* (suborder Ophionereidina). Within clade *j*, the Ophionereidinae (= genus *Ophionereis*) has ophiopsilid-like IFs (type B except for their large horizontal PEPs) and so far one hemieuryalid–*Ophioplocus januarii*—has been found to have the full complement of type B character states. However, IFs with single ambiguous or type B traits are present in the Amphilimnidae (adradial muscle attachment area of all species), Ophiolepididae (dental plate of a few species) and other hemieuryalids (dental plate of all *Ophiozonella* species). Furthermore, broad, though uncapped, teeth occur in some amphilimnids, hemieuryalids (certain *Ophiozonella* species and *Quironia johnsoni*) and ophiolepidids (certain *Ophiolepis* species). Whilst independent acquisition cannot be ruled out (but is highly improbable in the case of *O*. *januarii*), this scattering of type B traits and similarities across all four families of clade *j* again suggests the possibility that the type B state is apomorphic for the clade but has undergone extensive, though uneven, reversal in several separate lines. The mechanism underlying these evolutionary events may again be paedomorphosis, since the Hemieuryalidae and Ophiolepididae possess external skeletal characters considered to be paedomorphic [11], although another factor may be a clade *j*-specific propensity for type B trait reversal resulting in an “underlying synapomorphy” [58]. Since on present evidence type B character states are absent from the order Ophioleucida (families Ophioleucidae and Ophiernidae: clade *f* in Fig 10), we hypothesise that the type B IF is an apomorphy of order Amphilepidida (clade *e* in Fig 10) and therefore that the type A peristomial plates of the Ophiopsilidae reflect trait reversal rather than persistence of a plesiomorphic condition.

Type B character states are present in two other clades: (1) within suborder Ophiacanthina (clade *k* in Fig 10) the families Ophiopteridae and Clarkcomidae show a mosaic of IF traits; (2) in contrast to the other three uniformly type A families of suborder Ophiodermatina (clade *l* in Fig 10), all species of Ophiocomidae possess fully type B IFs, with the exception of the small fissiparous *Ophiocomella ophiactoides* [59,60], the type A PEPs of which are likely to be paedomorphic. This is the starkest manifestation of the incongruence that was first recognised by Matsumoto [21] and needs to be emphasised: the full complex of type B skeletal character states and their soft tissue correlates (including bursal diverticula, distal radial ligament, and the relative dimensions and disposition of the musculature) is present in the Ophiocomidae and in the phylogenetically remote families of suborder Gnathophiurina (clade *m* in Fig 10). What common selection pressures were responsible for this remarkable example of evolutionary convergence?

There is a strong correlation between bathome and IF type: without exception all clades with fully or mainly type B IFs (as shown in Fig 10) have a predominantly shallow bathymetrical range (i.e. intertidal– 200 m, as defined by Bribiesca-Contreras et al. [61]), whilst this applies to only two families with type A IFs (Ophiopezidae and Ophiodermatidae: see Figs S1 and S5 of [61]). The shallow-water distribution of type B families and suprafamilial clades is likely to be a derived trait [61] signalling a shift from deep sea (>200 m) to continental shelf and intertidal zones and thus indicating that type B IFs may have evolved in response to selective forces peculiar to shallow-water environments. This is endorsed by the distribution of IF types within suborder Gnathophiurina (clade *m* in Fig 10): all four families with type B IFs have a mainly shallow bathymetrical range, whereas the two families that have reverted to the fully A type state (Ophiothamnidae and Amphilepididae) are almost exclusively deep-sea ([61]; sources of information on Amphilepididae mainly holotype descriptions as referenced by Stöhr et al. [27]). The paedomorphic trait reversal undergone by the internal and external skeletons of these two families (discussed above) may thus be a consequence of their secondary return to the deep-sea bathome, with the further implication that type B IFs did not confer sufficient fitness advantage in that environment to elude paedomorphic trends.

The particular aspect(s) of life in shallow-sea environments to which type B IFs could be an adaptation is/are far from obvious, because for the past 160 years views on the functioning and functional morphology of the ophiuroid IF have been based largely on unsubstantiated speculation rather than empirical investigation. Since food items are conveyed by the buccal podia to the mouth through the star-shaped pre-oral cavity delimited by the IF and since the IF dilates to whatever extent is required to permit ingestion of food items and ejection of undigested material, it is self-evident that the IF is involved in alimentation. However, there is no unequivocal evidence to support the long-held assumption (disputed by some authors, e.g. [13,18,62]) that it is a “chewing” or “masticatory” apparatus (*pace* [15,21,24,63,64]), which, like the anatomical terminology that has reinforced it (“teeth”, “maxiller”, “jaws”), seems to have been originally inspired by little more than superficial resemblances. Such a role was not confirmed by a comprehensive analysis of the morphology, behaviour and physiology of an ophiodermatid (type A) IF ([65] and Candia Carnevali et al. unpublished data; see also [66]). There are no comparable data on any type B IF. It is, however, intriguing that, even taking into account the trophic versatility demonstrated by many ophiuroids [67–70], suspension feeding features prominently and consistently in all clades with type B IFs (as shown in Fig 10), but sporadically, or not at all, in those with type A IFs (see *inter alia* [37,63,68,69,71–73]). It has also been suggested that the capped teeth of type B IFs are an adaptation for the efficient comminution of material obtained by suspension or deposit feeding [36,64]. In view of the variation in IF morphology across the class, the functions and functioning of the IF are also likely to vary. It is possible, for example, that type B IFs evolved as a respiratory pump [25], the bursal diverticula within the external interradial muscles serving to optimise the flow of sea water through the genital bursae when the muscles contract and relax [40,43]. Such an innovation could have been an adaptive response to the greater metabolic requirements associated with life in higher energy and more mechanically challenging shallow-sea environments (and in the intertidal in particular), and indeed there is some evidence that ophiuroids with type B IFs tend to have higher respiratory rates than those with type A IFs (review in preparation).

## Conclusions

The peristomial plates of the ophiuroid interbrachial frame have taxonomic significance: their relative size and orientation represent stable characters that have diagnostic potential at familial and suprafamilial levels. They also have wider evolutionary significance in that their comparative morphology augments the evidence supporting the concept that two main IF types (designated ‘A’ and ‘B’ herein) are present in the Ophiuroidea. Current phylogenomic information and phylogenies derived from it imply that type A is ancestral and that type B evolved independently at least twice in the orders Amphilepidida and Ophiacanthida (clades *e* and *g* in Fig 10): the similarities between the IFs of amphilepididan families Ophiotrichidae, Ophiopholidae, Ophiactidae and Amphiuridae on the one hand and those of ophiacanthidan family Ophiocomidae on the other appear to represent a striking example of what Conway Morris has called “the repeated emergence of sophisticated adaptational complexes” [47]. It is, however, a highly unusual example of homoplasy in that the functional demands to which the morphological convergence was an evolutionary response are unknown.

The morphological differences between type A and B IFs indicate that the evolution of type B IFs was associated with a major qualitative or quantitative functional shift. The nature of that shift is completely obscure due to the dearth of fundamental knowledge about IF functioning and how this varies across the class. It is disconcerting that Warner’s comments on IFs made in 1982 [69]–“There are very few reports of the use of these complex structures.” and “The functional significance of the structural differences between species… is an unknown area.”—are hardly less true 36 years later. The present paper has concentrated on the endoskeleton of the IF and its systematic significance and has made little reference to its soft tissue components. It should be remembered that the IF is actuated by antagonistic sets of muscles; its joint motions are constrained by a complex system of ligaments, at least some of which consist of directly innervated mutable collagenous tissue [65]; it houses the circumoral nerve ring (and accompanying coelomic and haemal vessels) and water vascular ring (and attached polian vesicles), as well as the junctions between these rings and the proximal portions of the radial nerves and water vascular canals; it also accommodates the functionally enigmatic axial complex and, in each radius, the first two pairs of podia (i.e. the buccal podia) and the vessels connecting these to the water vascular ring [74–77]. Thus, despite current ignorance of IF functioning, its anatomy and anatomical relations by themselves intimate its central importance for ophiuroid biology. Comparative analyses of the ontogenetic development, behaviour, biomechanics and physiology of type A and B IFs would both fill a serious gap in our knowledge of the basic biology of the most speciose echinoderm class and improve understanding of the evolutionary processes responsible for the diversity and distribution of the extant ophiuroid fauna. We also hope that ophiuroid taxonomists will in future take more cognisance of the IF. As Fig 10 and S5 Table show, much more information is needed to permit a full appreciation of IF comparative morphology and its systematic potential. This could be facilitated by modern imaging techniques, such as X-ray micro-computed tomography, which, though compromising on microstructural detail, provide non-destructive means of determining many character states of the internal skeleton [31,78].

## Supporting information

S1 Table Geographical locations at which specimens of directly observed species were collected. Binomials and authorities in accordance with Stöhr *et al*. (2018) and family affiliations in accordance with O’Hara *et al*. (2018).
(XLSX)

S2 Table PEP character states of species that were examined directly. Binomials and authorities in accordance with Stöhr *et al*. (2018) and family affiliations in accordance with O’Hara *et al*. (2018). DD, disc diameter; n, number of peristomial plates from which measurements were taken; RL, relative length; RW, relative width. Where n>1, the values for RL and RW are means. For all species except *Ophiocomina nigra* one adult animal was examined; in the case of *O*. *nigra*, the mean values for RL and RW were derived from four animals all with disc diameter 11 mm.
(XLSX)

S3 Table PEP character states of species from families not included in S2 Table; information obtained from the literature. Binomials and authorities updated in accordance with Stöhr *et al*. (2018) and family affiliations in accordance with O’Hara *et al*. (2018). Information on only adult animals is included. *Because, according to Matsumoto (1917), *Histampica umbonata* lacks dental plates and because no dental plates are visible in Lyman’s illustration of *Ophiothamnus vicarius* (Lyman 1882: Pl. LXII, Fig 1), the width of the distal tooth base was used an approximate substitute for dental plate width.
(XLSX)

S4 Table Title PEP character states of additional species from families included in S2 Table; information obtained from the literature. Binomials and authorities updated in accordance with Stöhr *et al*. (2018) and family affiliations in accordance with O’Hara *et al*. (2018). Information on only adult animals is included. *The orientation of the PEPs is ambiguous in Matsumoto’s illustrations of *Ophiothela danae* and *Ophiactis profundi* (Matsumoto 1917: Pl. IV, Fig 8 and Pl. III, Fig 9 respectively).
(XLSX)

S5 Table Survey of all interbrachial frame character states. Binomials and authorities in accordance with Stöhr *et al*. (2018) and family affiliations in accordance with O’Hara *et al*. (2018).
(XLSX)

S1 Text References for S2–S5 Tables and their captions. (DOCX)
