# Supplementary material for: The peristomial plates of ophiuroids (Echinodermata: Ophiuroidea) highlight an incongruence between morphology and proposed phylogenies
Source: PLoS One. 2018 Aug 9;13(8):e0202046. doi: 10.1371/journal.pone.0202046 (PMC6084971; doi:10.1371/journal.pone.0202046)
Supplement: S1 Text — (DOCX) [file pone.0202046.s006.docx]

**S1 Text. References for S2-S5 Tables and their captions**.

Baker AN, Devaney DM. New records of Ophiuroidea (Echinodermata) from Southern Australia, including new species of *Ophiacantha* and *Ophionereis*. Trans R Soc S Austral. 1981;105(4): 155-178.

Bartsch I. Ophiuroidea (Echinodermata) from the Patagonian Shelf. Mitt hamb zool Mus Inst. 1982;79: 211-250.

Bartsch I. *Ophiambix meteoris* n.sp., ein neuer Schlangenstern aus der Iberischen Tiefsee. Spixiana. 1983a;6: 97-100.

Bartsch I. Ophiuroidea (Echinodermata) from the northeastern Atlantic deep sea. “Meteor” Forsch.-Ergebnisse, Reihe D. 1983b;36: 13-20.

Bartsch I. Notes on Ophiuroidea (Echinodermata) from the northeastern Atlantic Ocean. I. Ophiacanthidae. Spixiana. 1987;10: 115-130.

Berecoechea JJ. Alimentación en Ophiuroidea (Echinodermata) de Argentina: studios a partir del análisis de morfología y microestrctura de mandíbulas y dientes. Tesis di Licenciatura, University of Buenos Aires. 2014.

Brogger MI. Crecimento, reproducción y alimentación del ofiuroidea *Ophioplocus januarii* (Lütken, 1856) (Echinodermata: Ophiuroidea) en costas de Patagonia, Argentina. Ph.D. Thesis, University of Buenos Aires. 2010.

Brogger MI, Martinez MI, Cadierno MP, Penchaszadeh PE. Tooth microstructure and feeding biology of the brittle star *Ophioplocus januarii* (Echinodermata: Ophiuroidea) from northern Patagonia, Argentina. Rev Biol Trop. 2015;63 (Suppl. 2): 353-360.

Candia Carnevali MD, Bonasoro F, Wilkie IC. Structural and mechanical aspects of the mouth-frame of the brittlestar *Ophioderma longicaudum* (Retz.). In: David B, Guille A, Féral JP, Roux M, editors. Echinoderms through time. Rotterdam: Balkema; 1994. pp. 387-392.

Clark AM. Notes on the family Amphiuridae (Ophiuroidea). Bull Brit Mus (Nat Hist) Zoology. 1970;19(1): 1-81.

Clark HL. The sea-lilies, sea-stars, brittle-stars and sea-urchins of the South Australian Museum. Rec S Austral Mus. 1928;3(4): 361-482.

Dearborn JH, Hendler G, Edwards KC. The diet of *Ophiosparte gigas* (Echinodermata: Ophiuroidea) along the Antarctic Peninsula, with comments on its taxonomic status. Polar Biol. 1996; 16: 309-320.

Devaney DM. Studies on ophiocomids brittlestars. I. A new genus (*Clarkcoma*) of Ophiocominae with a reevaluation of the genus *Ophiocoma*. Smithsonian Contrib Zool. 1970;51: 1-41.

Devaney DM. Shallow-water echinoderms from British Honduras, with a description of a new species of *Ophiocoma* (Ophiuroidea). Mar Sci. 1974;24: 122-164.

Devaney DM. A review of the genus *Ophiomastix* (Ophiuroidea: Ophiocomidae). Micronesica. 1978;14: 273-359.

Gondim AI, Dias TLP, Christoffersen ML, Stöhr S. Redescription of *Hemieuryale pustulata* von Martens, 1867 (Echinodermata, Ophiuroidea) based on Brazilian specimens, with notes on systematics and habitat association. Zootaxa. 2016;3925(3): 341-360.

Koehler R. Ophiures de l’expédition du Siboga. 1ère Partie. Ophiures de Mer Profonde. Leiden: E.J. Brill; 1904.

Koehler R. Ophiures de l’expédition du Siboga. 2ème Partie. Ophiures Littorales. Leiden: E.J. Brill; 1905.

Liao Y. On *Amphilimna polyacantha* sp. nov. and the systematic position of *A.* *multispina* Koehler (Ophiuroidea). Chin J Ocean Limn. 1983;1: 177-182.

Lyman T. Report on the Ophiuroidea dredged by H.M.S. Challenger, during the years 1873-1876. Report on the scientific results of the voyage of H.M.S. Challenger. Zoology, Vol. 5. London: H.M. Stationary Office; 1882.

Madsen FJ. A review of the Ophioleucinae stat. rev. (Echinodermata, Ophiuroidea) with the erection of a new genus, *Ophiostriatus*. Steenstrupia. 1983;9: 29-69.

Martynov A. Reassessment of the classification of the Ophiuroidea (Echinodermata), based on morphological characters. I. General character evaluation and delineation of the families Ophiomyxidae and Ophiacanthidae. Zootaxa. 2010;2697: 1-154.

Martynov A, Ishida Y, Irimura S, Tajiri R, O’Hara T, Fujita T. When ontogeny matters: a new Japanese species of brittle star illustrates the importance of considering both adult and juvenile characters in taxonomic practice. PLoS ONE. 2015;10(10):e0139463. doi: 10.1371/journal.pone.0139463

Matsumoto H. A monograph of Japanese Ophiuroidea arranged according to a new classification. J Coll Sci Imp Univ Tokyo. 1917;38: 1-408.

Medeiros-Bergen DE. On the stereom microstructure of ophiuroid teeth. Ophelia. 1996;45: 211-222.

Mortensen T. Echinodermes du Maroc et de Mauritanie. Bull Soc Sci Nat Maroc. 1925;5: 178-187.

Murakami S. The dental and oral plates of Ophiuroidea. Trans R Soc New Zealand. Zoology. 1963;4(1): 1-48.

O’Hara TD, Harding C. Enigmatic ophiuroids from the New Caledonian region. Mem Mus Victoria. 2015;73: 47–57.

O’Hara TD, Stöhr S. Deep water Ophiuroidea (Echinodermata) of New Caledonia: Ophiacanthidae and Hemieuryalidae. In: Richer de Forge B, Justine JL, editors. Tropical deep-sea benthos, vol. 24. Mém Mus natl Hist nat. 2006;193: 33-141.

O’Hara TD, Stöhr S, Hugall AF, Thuy B, Martynov A. Morphological diagnoses of higher taxa in Ophiuroidea (Echinodermata) in support of a new classification. Eur J Taxon. 2018;416: 1-35.

Okanishi M, Fujita T, Maekawa Y, Sasaki T. Non-destructive morphological observations of the fleshy brittle star, *Asteronix loveni* using micro-computed tomography (Echinodermata, Ophiuroidea, Euryalida). ZooKeys. 2017;663: 1-19.

Okanishi M. Ophiuroidea (Echinodermata): systematics and Japanese fauna. In: Motokawa M, Kajihara H, editors. Species diversity of animals in Japan, diversity and commonality in animals. Japan: Springer; 2017. pp. 651-678.

Olbers JM, Samyn Y, Griffiths CL. New or notable records of brittle stars (Echinodermata: Ophiuroidea) from South Africa. African Nat Hist. 2015;11: 83-116.

Stöhr S. New records and new species of Ophiuroidea (Echinodermata) from Lifou, Loyalty Islands, New Caledonia. Zootaxa. 2011;3089: 1-50.

Stöhr S. Ophiuroid (Echinodermata) systematics – where do we come from, where do we stand and where should we go? Zoosymposia. 2012;7: 147-161.

Stöhr S, O’Hara TD, Thuy B. Global diversity of brittle stars (Echinodermata: Ophiuroidea). PLoS ONE; 2012;7(3): e31940. doi: 10.1371/journal.pone.0031940

Stöhr Tax S, Boissin E, Hoareau TB. Taxonomic revision and phylogeny of the *Ophiocoma brevipes* group (Echinodermata, Ophiuroidea), with description of a new subgenus (*Breviturma*) and a new species. Eur J Taxon. 2013;68: 1-26.

Stöhr S, Martynov A. Paedomorphosis as an evolutionary driving force: insights from deep-sea brittle stars. PLoS ONE. 2016;11(11):e0164562.doi:10.1371/journal.pone.0164562

Stöhr S, O’Hara T, Thuy B. World Ophiuroidea database; 2018. Accessed at: <http://www.marinespecies.org/ophiuroidea/aphia.php?p=taxdetails&id=244283>

Stöhr S, Muths D. Morphological diagnosis of the two genetic lineages of *Acrocnida* *brachiata* (Echinodermata: Ophiuroidea), with description of a new species. J Mar Biol Ass UK. 2009;90: 831-843.

Thomas LP. The systematic position of *Amphilimna* (Echinodermata: Ophiuroidea). Proc Biol Soc Wash. 1967;80: 123-130.

Thuy B, Stöhr S. A new morphological phylogeny of the Ophiuroidea (Echinodermata) accords with molecular evidence and renders microfossils accessible for cladistics. PLoS ONE. 2016;11(5): e0156140. doi: 10.1371/journal.pone.0156140

Wilkie IC. The systematic position of *Ophiocomina* Koehler and a reconsideration of certain interfamilial relationships within the Ophiuroidea. In: Jangoux M, editor. Echinoderms present and past. Rotterdam: Balkema; 1980. pp. 151-157.

Wilkie IC, Emson RH, Mladenov PV. Morphological and mechanical aspects of fission in *Ophiocomella ophiactoides*. Zoomorphology. 1984;104: 310-322.
